# [Retracted] Sulforaphane sensitizes human cholangiocarcinoma to cisplatin via the downregulation of anti‑apoptotic proteins

**DOI:** 10.3892/or.2024.8728

**Published:** 2024-04-01

**Authors:** Rokas Račkauskas, Dachen Zhou, Simonas Ūselis, Kęstutis Strupas, Ingrid Herr, Peter Schemmer

Oncol Rep 37: 3660–3666, 2017; DOI: 10.3892/or.2017.5622

Following the publication of the above article and a corrigendum that was published in October 2023 to address the issue of misplaced control β-actin western blots comparing between Figs. 3 and 4A (doi: 10.3892/or.2023.8646), an attentive reader drew to the authors' attention that the first author had apparently made additional unreported corrections to the revised version of Fig. 4 presented in the corrigendum. Although these image discrepancies did not alter the study's primary conclusions, they were such that they did cast doubt on the data's integrity. Consequently, the authors have decided to retract the paper and the Editor of *Oncology Reports* has agreed to the authors' request. The authors deeply regret any confusion or inconvenience this retraction may cause, and offer their sincere apologies to the Editor of *Oncology Reports* and the readership.

